# New Analysis Scheme of Flow-Acoustic Coupling for Gas Ultrasonic Flowmeter with Vortex near the Transducer

**DOI:** 10.3390/s18041151

**Published:** 2018-04-10

**Authors:** Yanzhao Sun, Tao Zhang, Dandan Zheng

**Affiliations:** School of Electrical and Information Engineering, Tianjin University, Tianjin 300072, China; zhengdandan@tju.edu.cn

**Keywords:** gas ultrasonic flowmeter, transducer, acoustic beam, velocity profile, ray tracing, wave acoustics, computational fluid dynamics

## Abstract

Ultrasonic flowmeters with a small or medium diameter are widely used in process industries. The flow field disturbance on acoustic propagation caused by a vortex near the transducer inside the sensor as well as the mechanism and details of flow-acoustic interaction are needed to strengthen research. For that reason, a new hybrid scheme is proposed; the theories of computational fluid dynamics (CFD), wave acoustics, and ray acoustics are used comprehensively by a new step-by-step method. The flow field with a vortex near the transducer, and its influence on sound propagation, receiving, and flowmeter performance are analyzed in depth. It was found that, firstly, the velocity and vortex intensity distribution were asymmetric on the sensor cross-section and acoustic path. Secondly, when passing through the vortex zone, the central ray trajectory was deflected significantly. The sound pressure on the central line of the sound path also changed. Thirdly, the pressure deviation becomes larger with as the flow velocity increases. The deviation was up to 17% for different velocity profiles in a range of 0.6 m/s to 53 m/s. Lastly, in comparison to the theoretical value, the relative deviation of the instrument coefficient for the velocity profile with a vortex near the transducer reached up to −17%. In addition, the rationality of the simulation was proved by experiments.

## 1. Introduction

Gas ultrasonic flowmeters with a small or medium diameter are widely used in natural gas metrology and process control areas. A very important category of ultrasonic flowmeter is based on the principle of transit time difference. Flow velocity is calculated using the time difference between upstream and downstream. From the review of ultrasonic flowmeter done by Lynnworth [1], Rajita [2], and Lansing [3], the working mechanism inside the sensor is an interaction process between the flow field and sound propagation. Moreover, the flowmeter performance is influenced by the flow-acoustic interaction process. So, the mechanism and details of flow-acoustic coupling are worth intensive study.

From the perspective of the flow field, great progress has been made in the ultrasonic flowmeter. On one hand, many scholars including Mandard [4], Peng [5], Marushchenko [6], and Hu [7] have conducted a lot of work on the variety of the flow velocity profile inside the sensor caused by pipeline configurations upstream, different acoustic path layouts, and the special geometry of sensor, etc. By improving the acoustic channel arrangement, using different velocity integral methods on the acoustic path, and other special calculation methods, the measurement performance of the flowmeter is improved [8]. On the other hand, in a pipe with a small or medium size, the flow field disturbance produced by the transducer installation position has also attracted more attention. The flow field distortion caused by the transducer recess or protrusion is reported in ASME PTC 18-2002 [9]. From 1996 to 2014, in order to analyze the flow field disturbance caused by the transducer installation position, simulations or experiments were executed by VOSER [10], LOWFLL [11], Raisutis [12], Zheng [13], and Guo [14]. In References [10,11,12,13,14], it was found that an asymmetrical velocity profile is found on the acoustic path. Also, the velocity profile with complex shapes was shown in the transducer concave cavity. Besides, the reflux length on the acoustic path was found to increase with the Reynolds number in a laminar flow regime. As a result, the error of the flow measurement was determined.

The studies described above are mainly from the perspective of the flow field. However, the working mechanism in an ultrasonic flowmeter is a flow-acoustic coupling process. Sound propagation in fluid has also been analyzed by researchers employing the ray acoustics. In 2002 to 2016, the ray tracing method was used by Koechner [15], Bezdek [16], Tezuka [17], Iooss [18], Kupni [19], Li [20], and Zheng [21] ultrasonic flowmeter studies. It was found that the sound propagation path deviates from the straight line because of the flow profile. Due to the ray trajectory (i.e., sound beam) offset, the sound pressure is 0.45 times that of the velocity 0 m/s, under the conditions of turbulent velocity profile, velocity 30 m/s, and temperature 20 °C. The ray trajectory offset increases with the increase of the flow velocity. Also, the ray trajectory offset downstream is greater than the upstream situation. It is clearly seen that the velocity profile used for study is mainly the turbulent profile; the complex profile is less mentioned [20,21].

Ray acoustics are derived from wave acoustics. They are an approximate solution for wave front tracing under high-frequency sound waves. The ultrasonic flowmeter is also studied by scholars from the perspective of wave acoustics. From 1996 to 2016, acoustic field studies with wave acoustics using the finite element method (FEM) or semi analytical approach inside a sensor was implemented by Eccardt [22], Willatzen [23,24,25], Chen [26] and Luca [27,28]. The results are presented as follows. The factors such as transducer axis parallel to the pipe axis, different sizes of transducer, profile type (uniform, quadratic or cubic function, laminar flow, and turbulence) are included. A significant difference of beam drift existed between the laminar flow and turbulent flow profile. Measurement deviation of the flowmeter was significantly different along with the changes of velocity profile and velocity. From the above research, the background flow fields were used to elucidate the sound propagation inside the sensor, which can be uniform, turbulent, or asymmetric flow profiles according to the formula. The sound propagation in a complex flow profile with vortex near the transducer still requires further study. In 2016, a numerical method based on wave acoustics (linear Euler equation) was designed by Luca [27]. The sound pressure on receiver was obtained with eight velocities in the range of 0 m/s~30 m/s, using the velocity profile obtained from the CFD. It was suggested by Luca that the influences of the transducer position on the acoustic field and measurement need to be further studied. In addition, the transient calculation complexity of the wave acoustics and three-dimensional geometry was also pointed out. Furthermore, the complexity of the full time-domain computing necessary for the wave equation was also reported in another article by Luca (2016) [28].

From the literature investigation, the main findings of the ultrasonic flowmeter studies are as follows. From the perspective of the flow field, great progress has been made. However, to the author’s knowledge, the study of flow-acoustic interaction using ray acoustics (or wave acoustics) with the complex flow field from CFD is still need to be studied, because such studies are mainly conducted with uncomplicated velocity profiles including uniform, laminar, or turbulent, etc. Besides, the mechanism and details of flow-acoustic interactions inside an ultrasonic flow sensor also requires further study.

The main contributions in this paper are as follows. Firstly, in order to extend the existing theory to the field ahead of the transducer, a new hybrid scheme is proposed to deal with the flow-acoustic coupling problems inside the sensor. In this scheme, a new step-by-step (i.e., sequential) method for flow-acoustic coupling analysis is also proposed. The mechanism and details of flow field changes caused by transducer position, as well as its effect on the sound propagation, receiving, and flowmeter performance are discussed. Secondly, the advantages of three methods—fluid mechanics, wave acoustics, and ray acoustics—are comprehensively utilized. Lastly, the analysis in this work provides insight into flow-acoustic coupling with the aim of improving the ultrasonic flowmeter performance.

The following work is conducted in this article. Firstly, according to principle of flowmeter, a new hybrid scheme is designed. Then, the SST turbulence model, the linear potential flow equation, and the ray tracing equation are solved using a proven FEM scheme. Also, the representative objects or locations are chosen for flow field and acoustics analyses. After that, the flow pattern features inside the sensor are obtained. Lastly, the sound propagation and receiving features, as well as the flowmeter performance influenced by flow pattern, are discussed in detail. In addition, an experiment is conducted to validate the new hybrid scheme.

## 2. New Hybrid Scheme and Numerical Realization for the Ultrasonic Flowmeter

### 2.1. Principle of the Ultrasonic Flowmeter

**Figure 1:**
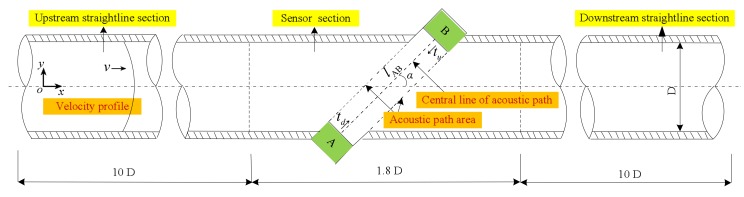
Schematic of the ultrasonic flowmeter.

Figure 1 depicts schematic of the ultrasonic flowmeter. In Figure 1, the fluid passes through the sensor from left to right. The transducer A and B are of the reciprocity type. The ultrasonic wave can be transmitted and received alternately from A or B. Then, the flow velocity can be calculated by measuring the transit time upstream and downstream, as follows:

(1) $$\left\{ \begin{array}{l} v=\frac{l(t_{u}-t_{d})}{2t_{u}t_{d}\cos\alpha }\\ t_{d}=\frac{l}{\left(c_{0}+v\cos\alpha \right)}\\ t_{u}=\frac{l}{\left(c_{0}-v\cos\alpha \right)} \end{array}\right.$$

where the parameters $t_{d}$, $t_{u}$, l, $c_{0}$ and α are downstream transit time, upstream transit time, sound path length, sound speed, and sound path angle (the angle between acoustic path and the pipeline axis) respectively. Here α = 45°.

Based on the measuring principle, analyses are as follows.

- The parameters that impact the flowmeter performance are l, α, $c_{0}$, and the flow velocity (or velocity profile), respectively.
- Although the influence of sound pressure on measurement is not shown in (1), the sound propagation is an interaction process between flow and acoustics. It is known that sound signals from the transmitter are passed through velocity profile first, then received by the receiver, and finally transformed into electrical signals. The sound features play an important role in the design of the transducer and digital processing circuit. Therefore, the influence of the velocity profile on the acoustic pressure and transit time needs to be taken into consideration.

### 2.2. New Hybrid Scheme

**Figure 2:**
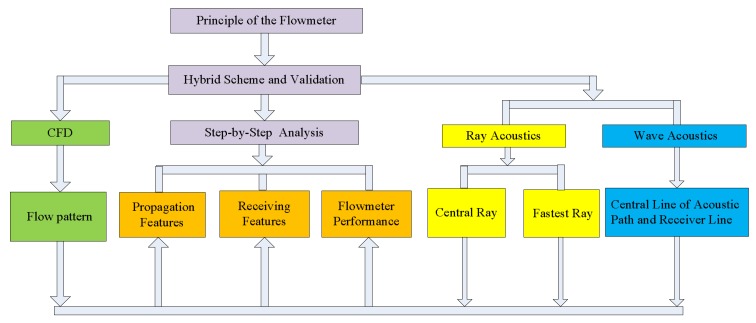
New hybrid scheme of flow-acoustic coupling study for a gas ultrasonic flowmeter.

Based on the principle of the ultrasonic flowmeter described above, a new hybrid scheme is developed, as shown in Figure 2.

- In the scheme, the advantages of the three theories are comprehensively utilized including CFD, ray acoustics, and wave acoustics. In the implementation, the SST turbulence model, the ray tracing equation, and the linear potential flow equation are solved by using the FEM. Also, three simple examples with analytical solutions are used for the scheme verification. A new “step-by-step approach” is used for the combination of the three theories.
- Then, some representative locations or objects such as the crosssection of the sensor, central line of the acoustic path, receiver line, central ray, and fastest ray are chosen for analyses. On those locations, the flow patterns are obtained such as flow velocity profile, velocity distribution, upstream and downstream, velocity profile partition, and vortex intensity, etc.
- After that, in accordance with the flow patterns, the acoustic propagation features, for instance, ray trajectory, slope along trajectory, offset al.ong trajectory, ray trajectory length, and acoustic pressure on the central line of the sound path, are obtained using the central ray and acoustic beam. Moreover, the receiving features, for example, deflection angle, offset, sound pressure on the receiver, and transit time influenced by flow pattern, are also discussed in detail.
- Finally, the instrument coefficient of I regarding the flowmeter performance is also analyzed.

### 2.3. Numerical Method

The information about acoustics and flow field is difficult to obtain by means of experiments or analytical methods at present. However, it can be obtained by the numerical method. When solving the wave acoustic field, the FEM software COMSOL is used. The four flow-acoustic coupling equations (including the linear potential flow equation, linear Euler equation, linear N-S equation, and convection wave equation provided by the software) that were derived by basic equations (such as the continuity equation, momentum equation, and state equation) are comprehensively compared. In order to save cost for solving the wave acoustic equation in ultrasonic frequency, the application condition of the flowmeter is simplified. Conditions such as 293 K, 0.1 MPa, inviscid fluid, and air are adopted. Besides, for the purpose of reducing the number of dependent variables, the linear potential flow equation is chosen. The frequency domain form is as below:

(2) $$-\frac{\rho_{0}}{c_{0}^{2}}i\omega \left(i\omega \phi +v\cdot \nabla \phi \right)+\nabla \cdot \left[\rho_{0}\nabla \phi -\frac{\rho_{0}}{c_{0}^{2}}\left(i\omega \phi +v\cdot \nabla \phi \right)v\right]=0$$

Here, $\rho_{0}$, v, and ϕ are the fluid density, flow velocity vector, and the acoustic velocity potential, respectively.

To reduce the computing cost of the wave equation in the time domain, the ray acoustic theory is adopted. The sound propagation feature and transit time can be obtained by using (3):

(3) $$\left\{ \begin{array}{l} \omega =c_{0}\left|k\right|+v\cdot k\\ \frac{dq}{dt}=\frac{\partial \omega }{\partial k}\\ \frac{dk}{dt}=\frac{\partial \omega }{\partial q}\\ k=k\frac{L_{0}}{\left|L_{0}\right|} \end{array}\right.$$

where ω, k, k, t, q, and $L_{0}$ are the acoustic angular frequency, wave vector, wave number, time, ray position vector, and ray direction vector, respectively.

**Figure 3:**
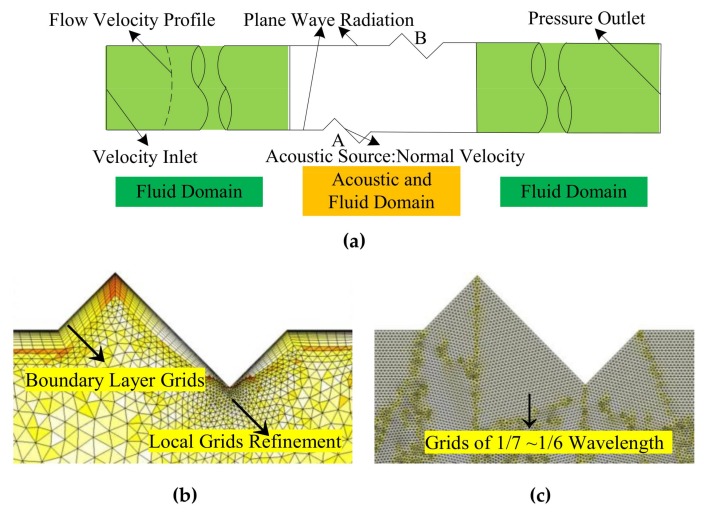
Physical field and grids settings. (**a**) Physical field settings. (**b**) Grid settings for the fluid domain. (**c**) Grid settings for the acoustic domain.

When solving Equations (2) and (3) with the FEM, the velocity profile, geometry and grid, boundary conditions, and solution methods are specified. Note that the following schemewas discussed to meet the requirements of calculation precision. So, it is not necessary to explain the reasons for choosing the set parameters in detail. Figure 3 shows the sketch of the physical field and grid settings.

For the background flow velocity, two types of profile are chosen, with or without reflux. Firstly, the uniform flow velocity profile (v = constant, hereinafter referred to as the U profile) and the turbulent velocity profile (T profile) [20,21] belong to the “no reflux” velocity profile. Secondly, the velocity profile obtained by CFD simulation considering the protrusion and recess of the transducer (PR profile) belongs to the ‘‘with reflux’’ situation. When solving the flow field, the following parameters such as the SST turbulence model, T profile for the inlet velocity, turbulent kinetic energy, specific dissipation rate, and pressure outlet are selected. The nine velocities (using the average flow velocity of the T profile as a benchmark), including 0 m/s, 0.55 m/s, 3.24 m/s, 8.04 m/s, 12.82 m/s, 22.35 m/s, 31.86 m/s, 42.41 m/s, and 52.95 m/s, are used for the inlet velocity.

The geometry and grids are as follows. Firstly, the geometry parameters are two-dimensional, with a pipe diameter of *D* = 100 mm, sensor section length of 180 mm, and straight pipe of 10 *D* upstream (or downstream) of the sensor. The transducer is the piston type with a diameter of 25 mm, and its center is aligned with the inner pipe wall. Secondly, the solving domain is discretized. For the acoustic field, the grid used in the sensor section is 1/7~1/6 of an acoustic wavelength at a frequency of 120 KHz. The grids for fluid mechanics are used in the remaining domains [29]. The “boundary layer grids” and “local grids refinement” are also used on the pipe wall and transducer surface, respectively. The domain discrete mode is freedom triangular, and the grid number is 306 thousand, as shown in Figure 3.

The boundary conditions for Equations (2) and (3) are as follows (shown in Figure 3). Firstly, for the wave Equation (2), the boundary condition at the acoustic source is the normal velocity, and the other boundary condition of the sensor is the plane wave radiation. Secondly, for the Equation (3), based on the theory that any complex sound source can be composed by many point sources, a point source is used to simplify the analysis. The point source is placed at the center of the piston, which emits one ray (the central acoustic ray) or more than one ray (ray beam), respectively. The ray frequency, ultrasonic wavelength, and the point source radius are 120 KHz, 2.86 mm, and 0.01 mm respectively. For the ray beam, in order to get enough density of rays to track the sound propagation, the parameters such as the conical angle of pi/3, rays of 61, and angle of pi/180 among the rays are adopted.

Finally, the solver settings are addressed. Firstly, the steady-state solver and the direct coupling method are used to solve Equation (2). Secondly, the ray tracing with generalized α solver is selected to solve Equation (3). For the time step, 0.02 μs is chosen for the flow velocity of 0.55 m/s, and 0.05 μs for the rest of the velocities. The time step is met with the specification of JJG 1030-2007, in that the error is less than 1% for the general ultrasonic flowmeter.

### 2.4. Verification of the Numerical Method

When the numerical method is used, it is necessary to verify the correctness. Here, three examples with analytical solutions are used in the free acoustic field, which include a point source in a stationary fluid (with uniform flow) and a piston source in a stationary fluid. With a stationary fluid and uniform flow, the radiated sound pressure of the point source [30] is shown by (4).

(4) $$\left\{ \begin{array}{l} p=j\frac{\rho_{0}c_{0}kr_{0}V_{a}}{r\sqrt{1+{\left(kr_{0}\right)}^{2}}}e^{j\left(2\pi ft-kr\right)}\\ \;=j\frac{\rho_{0}c_{0}kr_{0}V_{a}}{r\sqrt{1+{\left(kr_{0}\right)}^{2}}}e^{-k_{2}r}e^{j\left(2\pi ft-k_{1}r\right)}\\ k=\frac{2\pi f}{c_{0}}\\ k_{1}=\frac{2\pi f}{\left(c_{0}+v\cos\theta \right)}\\ k_{2}=\frac{v\cos\theta }{\left(\left(c_{0}+v\cos\theta \right)r\right)} \end{array}\right.$$

where $k_{1}$ and $k_{2}$ are two components of k. $V_{a}$, $r_{0}$, and r are the vibration velocity amplitude, radius of point source, and propagation distance, respectively. θ denotes the angle between the direction of v and “the link line of the point source and the sound field point”. 

The theoretical value [31] of the acoustic pressure amplitude on transducer axis is shown in (5):

(5) $$p_{a}\approx 2\rho_{0}c_{0}V_{a}\frac{ka^{2}}{4z}=\frac{\rho_{0}c_{0}}{2z}ka^{2}V_{a}$$

where z and a are axial coordinates on the transducer axis and the circular radiation surface radius of the piston, respectively.

The relative error δ between the simulation and analytical solution is obtained by using (6):

(6) $$\delta =\frac{Lp_{2}-Lp_{1}}{Lp_{1}}\times 100\%$$

where $Lp_{2}$ and $Lp_{1}$ are the sound pressure level (SPL) of the simulation and analytical solution, respectively. The relative error is less than 1%. Ultimately, the numerical method is verified.

## 3. Results and Discussion

In this section, the new “step-by-step (sequential) approach” is introduced in detail. Then, by using this approach, the flow field with a vortex near the transducer, and its influence on sound propagation, receiving, and flowmeter performance are analyzed in depth, as well as compared with the results from the U and T profiles. 

### 3.1. Step-By-Step Approach for Flow-Acoustic Coupling Analysis inside the Sensor

In this subsection, three aspects of the “flow field”, the “CFD and ray acoustics”, and the “CFD, ray acoustics, and wave acoustics” are included in the “hybrid scheme” by using a step-by-step approach. Then, in the results analysis, a new analytical method for the central ray and the fastest ray is proposed.

#### 3.1.1. Flow Field Obtained Only by CFD and Comparison

**Figure 4:**
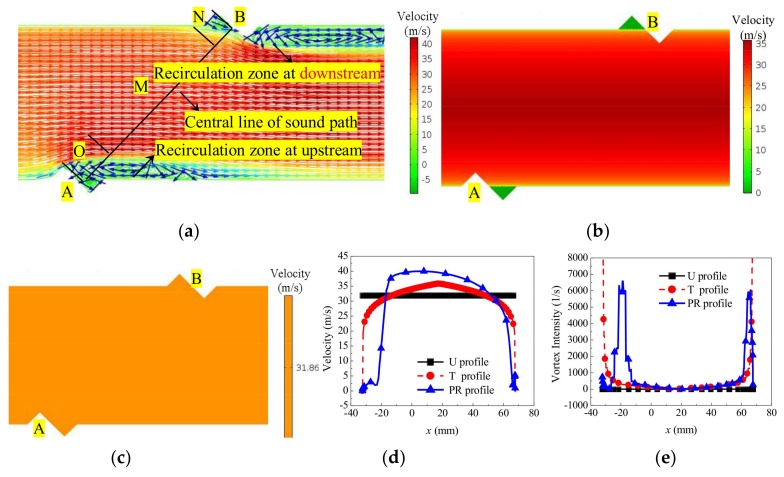
Background flow field inside the sensor (v = 31.86 m/s). (**a**) Velocity of the PR profile. (**b**) Velocity of the T profile. (**c**) Velocity of the U profile. (**d**) Velocity on the central line of the sound path. (**e**) Vortex intensity on the central line of the sound path.

The flow field is first discussed. Here, the cross-section of the sensor, and the central line of the sound path (line $l_{\mathrm{AB}}$) are chosen for flow field observation. Figure 4a expresses the PR velocity profile. Figure 4b,c gives the T and U profile for comparison. Figure 4d,e gives the velocity and vortex intensity distribution on line $l_{\mathrm{AB}}$ for the three profiles, respectively.

From Figure 4, conclusions are as follows.

- When the fluid is flowing through the transducer, similar to the backward-facing step flow, the vortex and recirculation zone are generated due to the throttling effect caused by the transducer. In the backflow zone, a negative velocity appears near the transducer, which is an important factor affecting the performance measure.
- The vortex intensity J is used to describe the velocity profile containing the vortex, as shown in (7).
  (7) $$J=\iint_{\sigma }\omega_{n}d\sigma$$
  where dσ is the fluid element, and $\omega_{n}$ is the normal component of the rotating angular velocity ω for the fluid element.
- According to the vortex intensity and velocity distribution on line $l_{\mathrm{AB}}$, three zones are formed (Figure 4a), including the upstream O, central M, and downstream N zones. The lengths of the three zones on the line $l_{\mathrm{AB}}$ are not sensitive to the velocity change by the velocity or vortex intensity statistics in the range of 0.55 m/s–52.95 m/s. For the PR profile, the approximate scope of the O, M and N on line $l_{\mathrm{AB}}$ are −36 mm–10 mm, −10 mm–60 mm, and 60 mm–70 mm, respectively, based on the x coordinate (Figure 4d,e). In the O and N zones, for the PR profile, sudden changes of flow velocity and vortex intensity are exhibited, and the trend and value are different. In the M zone, the velocity distributions of the three profiles are significantly different. The descending order of velocity is as follows: PR, T, and U profiles. Besides, the vortex intensity tends to zero in the M zone, and the sudden change is not shown.
- In addition, the velocity and vortex intensity distribution on the sensor cross-section are different. Compared with the U and T profile (without the recirculation zone), the asymmetry feature appears on the PR profile due to the recirculation zone.

In brief, the velocity profile is influenced by the vortex due to the throttling action of the transducer. Furthermore, from the relationship of v⋅∇ϕ and v⋅k, as shown in Equations (2) and (3), the sound field will be affected by the velocity profile.

#### 3.1.2. Combination of CFD and Ray Acoustics

**Figure 5:**
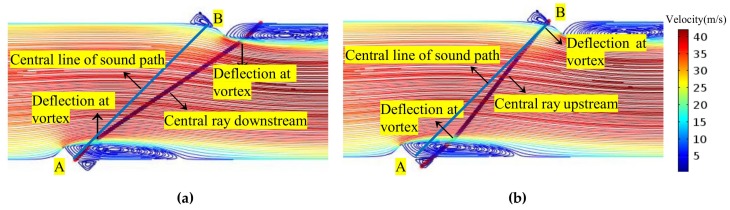
Central acoustic ray in flow field (the PR profile, v = 31.86 m/s). (**a**) Downstream; (**b**) Upstream.

In the background flow field, a ray (the central ray) is chosen that is located at the midpoint of the transmitter surface, and the ray launches perpendicular to the transducer surface. In this way, CFD and the ray acoustics are coupled. Figure 5 shows the ray trajectory influenced by the flow field both downstream and upstream. From Figure 5, the propagation and receiving features of the acoustic ray influenced by the flow field can be analyzed. 

#### 3.1.3. Combination of CFD, Ray Acoustics, and Wave Acoustics

**Figure 6:**
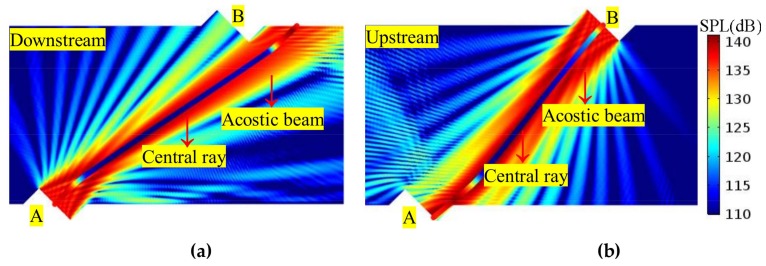
The central ray and the acoustic beam in the flow field (v = 30 m/s, the PR profile). (**a**) Downstream. (**b**) Upstream.

In the background flow field, through the central ray and the acoustic beam, the ray acoustics can be connected with the wave acoustics. In this way, advantages of both theories can be utilized comprehensively. On one hand, using the ray acoustics, the time domain features of sound propagation can be obtained. On the other hand, utilizing the wave acoustics, the propagation and receiving features of the acoustic beam generated by the piston source can also be expressed. Figure 6 depicts the superposition of the central ray and the acoustic beam. It can be seen that the propagation and receiving features of the acoustic beam are well indicated by the central ray.

**Figure 7:**
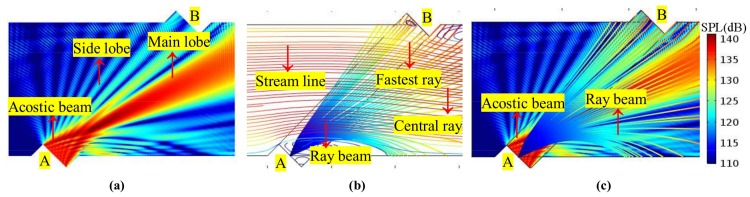
Relations among the acoustic beam, ray beam, central ray, and fastest ray in the flow field (v = 50 m/s, PR profile, downstream). (**a**) Acoustic beam. (**b**) Ray beam and streamline. (**c**) Ray beam and acoustic beam.

Although the central ray can be used for time domain analysis, the transit time is determined by multiple rays (i.e., a ray beam) for several reasons. Figure 7 shows the relations of the acoustic beam, ray beam, central ray, and fastest ray in the flow field. It can be seen that, firstly, the center ray deviates from receiver (i.e., leaving the receiver) when the flow velocity grows larger (for example, v > 30 m/s). So, the transit time cannot be derived from the central ray. Secondly, the relationship between the central ray and the acoustic beam (which is used in wave acoustics) is considered. That is, when the flow velocity grows larger, the main lobe deviates from the receiver, and the side lobe remains on the receiver (see Figure 6 and Figure 7). For these two reasons, the fastest ray in the ray beam (that is, the first ray to reach the receiver) is chosen for the transit time determination.

#### 3.1.4. Analytical Method for the Central Ray and the Fastest Ray

**Figure 8:**
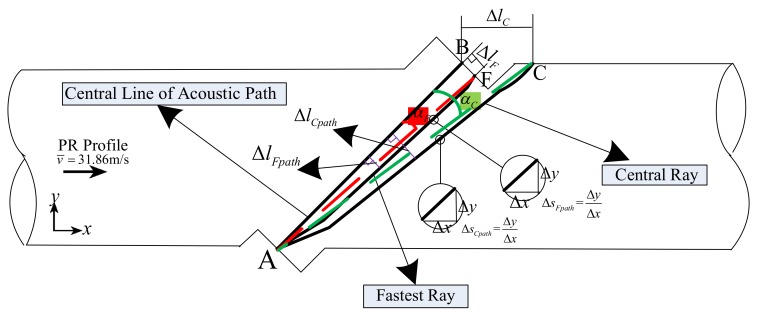
Sketch of the dimensionless parameter used in the ray result analysis.

In order to clearly show the influence of the flow field on the central ray and the fastest ray, dimensionless parameters are defined. Figure 8 depicts the sketch of the dimensionless parameter used in the ray result analysis. 

Firstly, the ray propagation parameters are described. The path offset $\Delta l_{path}$, and the path slope $\Delta s_{path}$ are used for the propagation path analysis of the central ray and fastest ray, respectively. The path offset $\Delta l_{path}$ is defined as the vertical distance between each point on the ray trajectory and the line $l_{\mathrm{AB}}$. Another parameter, $\Delta s_{path}$, is defined as the slope between every two adjacent discrete points along the ray trajectory. The subscripts C and F represent the central ray and the fastest ray, respectively.

Secondly, the ray receiving parameters are also defined. Here, Δl, α, and $l_{path}$ denote the receiving offset, ray path angle, and ray path length, respectively. Δl is the distance between B$\left(x_{B},y_{B}\right)$ and C$\left(x_{C},y_{C}\right)$ (or F$\left(x_{F},y_{F}\right)$) for the central ray (or fastest ray). α is the angle between line $l_{AB}$ and $l_{AC}$ (or $l_{AF}$) for the central ray (or fastest ray).

These parameters are obtained by using (8):

(8) $$\left\{ \begin{array}{l} l_{path}=\sum_{i=1}^{N-1}\sqrt{{\left(x_{i+1}-x_{i}\right)}^{2}+{\left(y_{i+1}-y_{i}\right)}^{2}}\\ \Delta l_{path}=\frac{\left|\beta x_{i}+\eta y_{i}+\zeta \right|}{\sqrt{\beta^{2}+\eta^{2}}}\\ \Delta s_{path}=\frac{y_{i+1}-y_{i}}{x_{i+1}-x_{i}}\\ t=\left(N-1\right)\cdot \Delta t\\ \Delta l_{C}=\sqrt{{\left(x_{C}-x_{B}\right)}^{2}+{\left(y_{C}-y_{B}\right)}^{2}}\\ \Delta l_{F}=\sqrt{{\left(x_{F}-x_{B}\right)}^{2}+{\left(y_{F}-y_{B}\right)}^{2}}\\ \alpha =\left|\frac{s_{2}-s_{1}}{1+s_{1}\cdot s_{2}}\right|\\ s_{2}=\frac{y_{C}-y_{A}}{x_{C}-x_{A}}\left(or\;s_{2}=\frac{y_{F}-y_{A}}{x_{F}-x_{A}}\right)\\ \alpha_{F}=\arcsin\left(\frac{\sqrt{{\left(x_{F}-x_{B}\right)}^{2}+{\left(y_{F}-y_{B}\right)}^{2}}}{\sqrt{{\left(x_{F}-x_{A}\right)}^{2}+{\left(y_{F}-y_{A}\right)}^{2}}}\right) \end{array}\right.$$

Here, $\left(x_{i},y_{i}\right)$ and $\left(x_{i+1},y_{i+1}\right)$ are the coordinates of the two adjacent points on the ray trajectory. $\left(x_{A},y_{A}\right)$ are the coordinates of midpoint A of transducer A. β, η, and ζ denote coefficients; β = 1, η = −1, ζ = −0.018. $s_{1}$, $s_{2}$ are slopes of $l_{AB}$ and $l_{AC}$, respectively; $s_{1}$ = 1. n is the last discrete point number on the ray trajectory.

### 3.2. Acoustic Propagation Features Influenced by the Flow Field inside the Sensor

In this subsection, by using the central ray (based on the ray acoustics), acoustic beam (based on the wave acoustics), and central line of the acoustic path, acoustic propagation features influenced by the flow field (from CFD) are analyzed. 

#### 3.2.1. Propagation Features Obtained by the Central Ray

**Figure 9:**
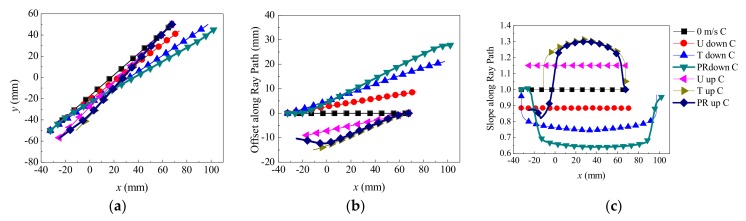
Propagation features of the central ray in different flow patterns (v = 31.86 m/s). (**a**) Central ray propagation path. (**b**) Offset al.ong the central ray ($\Delta l_{Cpath}$ ). (**c**) Slope along the central ray ($\Delta s_{Cpath}$ ).

First of all, propagation features of the central ray are analyzed. Figure 9 illustrates the ray propagation path, the path offset $\Delta l_{Cpath}$, and the path slope $\Delta s_{Cpath}$, respectively. In Figure 9, four sets of parameters including (Stationary, U, T, PR profile), (downstream, upstream), (v), and (propagation path, $\Delta l_{Cpath}$, $\Delta s_{Cpath}$) are shown. The “down”, “up”, and “C” in the legend of Figure 9 represent the downstream, upstream, and central ray, respectively.

According to Figure 9, the features of $\Delta l_{Cpath}$ and $\Delta s_{Cpath}$ are discussed:

- When v = 0 m/s, the ray trajectory upstream (or downstream) is overlapped with the line $l_{\mathrm{AB}}$. $\Delta l_{Cpath}$ = 0, $\Delta s_{Cpath}$ = 1.
- When v increases, the central rays for all velocity profiles deviate from the line $l_{\mathrm{AB}}$, and are moved toward the flow direction. The $\left|\Delta l_{Cpath}\right|$ and $\left|\Delta s_{Cpath}\right|$ are increased as the v increases. Downstream, $\Delta l_{Cpath}$ > 0 and $\Delta s_{Cpath}$ < 1, whereas upstream, $\Delta l_{Cpath}$ < 0 and $\Delta s_{Cpath}$ > 1.
- When v is equal, the $\Delta l_{Cpath}$ and $\Delta s_{Cpath}$ are different for each flow velocity profile.

From Figure 9, it can be seen that the segmented feature is obvious on the ray trajectory due to the velocity profile (see Figure 5), described as follows:

- The vortex near the transducer plays a big role in the ray deflection. When the central ray comes out of the vortex zone upstream, apparent deflection occurs. After entering into the central zone, deflection is mitigated. Finally, when passing through the vortex zone downstream, an obvious deflection appears again.
- The ray deflection degree (change of $\Delta l_{Cpath}$ and $\Delta s_{Cpath}$) in the vortex upstream is greater than that in the downstream section. The deflection degree is proportional to the vortex intensity in the flow field where the ray passes through.
- The propagation features of the central ray in the PR profile is significantly different from the U and T profiles, because of the vortex near the transducer.

#### 3.2.2. Propagation Features Obtained by the Acoustic Beam 

**Figure 10:**
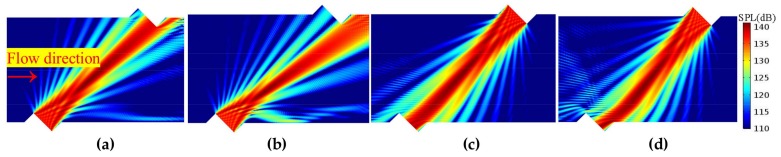
Acoustic beam for the PR profile (flow direction is from left to right). (**a**) Downstream, v = 12.82 m/s. (**b**) Downstream, v = 31.86 m/s. (**c**) Upstream, v = 12.82 m/s. (**d**) Upstream, v = 31.86 m/s.

**Figure 11:**
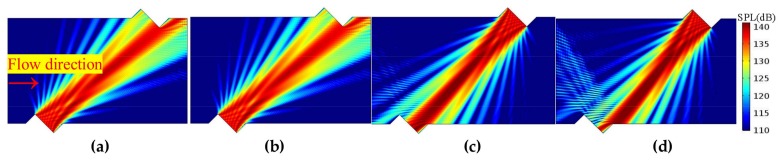
Acoustic beam for the T (i.e., turbulent) profile (flow direction is from left to right). (**a**) Downstream, v = 12.82 m/s. (**b**) Downstream, v =31.86 m/s. (**c**) Upstream, v = 12.82 m/s. (**d**) Upstream, v = 31.86 m/s.

**Figure 12:**
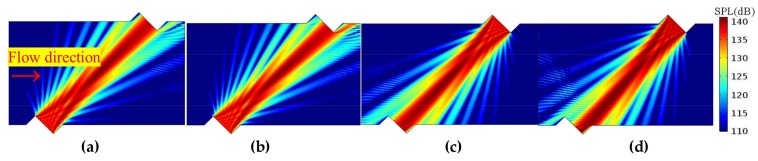
Acoustic beam for the U (i.e., uniform) profile (flow direction is from left to right). (**a**) Downstream, v = 12.82 m/s. (**b**) Downstream, v = 31.86 m/s. (**c**) Upstream, v = 12.82 m/s. (**d**) Upstream, v = 31.86 m/s.

Firstly, on the sensor cross-section, the propagation and receiving features of the acoustic beam are analyzed. Figure 10, Figure 11 and Figure 12 show the sound beam in different flow patterns. The following phenomena are discovered:

- When v increases, at the downstream (or upstream) situation, the sound beam is deflected to the flow direction for each profile. As a result, the main lobe having the most energy deviates from the center of the receiver, whereas the side lobe is gradually moved to the receiver.
- When v is equal, the acoustic beams downstream and upstream are different for the same profile. Moreover, the acoustic beams downstream (or upstream) are also different for different profiles.
- When v increases (e.g., v > 22.35 m/s), compared with the profiles with non-recirculation, the acoustic beam deflects (or bends) more significantly due to the recirculation zone.

The above phenomena are consistent with the results obtained from the central ray.

**Figure 13:**
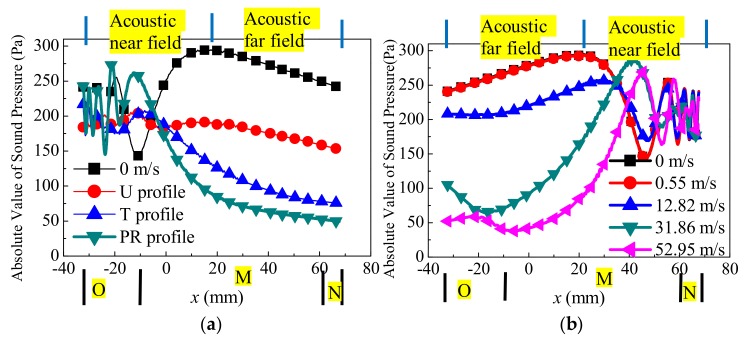
Propagation features of the acoustic beam on the central line of the acoustic path. (**a**) Downstream propagation (v = 31.86 m/s, different velocity profile). (**b**) Upstream propagation (different flow velocity, PR profile).

Secondly, except for the sensor cross-section, on the central line of acoustic path, the sound pressure distributions are also analyzed. More importantly, the partition relations between the velocity profile (Figure 4a,d,e) and acoustic field (near field and far field of the piston transducer) are also interpreted. Figure 13a shows the sound pressure distribution for different profiles on line $l_{\mathrm{AB}}$. Analyses are as follows.

- The O zone and acoustic near field. The far-near field demarcation $z_{g}$ of the piston transducer is shown in (9).
  (9) $$z_{g}=\frac{a^{2}}{\lambda }$$
  Here, λ is the acoustic wave length. It can be seen that the near field is located within a distance of 55mm on line $l_{\mathrm{AB}}$. The undulations of the acoustic pressure amplitude are observed in the near field. When v increases, the near field is “shortened” compared with the “v = 0 m/s” case. This is because the center axis of the acoustic beam shifts away from the line $l_{\mathrm{AB}}$ and deflects to the flow direction.
- The M zone and acoustic near field (or acoustic far field). The distance within 55 mm is the near field, and the rest is the far field. In the far field, the sound pressure amplitude decreases as the distance increases. The sound pressure is different for each profile. The sound pressure also decreases as the velocity increases.
- The N zone and acoustic far field. The phenomena appearing in this zone are similar to that of the “M zone and acoustic far field” described above.

Figure 13b shows the sound pressure distribution of five velocities on line $l_{\mathrm{AB}}$ for the PR profile in the upstream situation. Based on the partition relation between the flow field and acoustic field, analyses are as follows:

- The O and N zones. In the upstream situation (contrary to the downstream), the acoustic beam passes successively through the N, M, and O zones. The sound pressure distribution in the N zone upstream is similar to that of the O zone downstream. Note that, in the O zone, the sound pressure abnormally increases with increasing distance due to the vortex near the transducer (Figure 4).
- The M zone and acoustic far field. In the M zone that overlaps with the acoustic far field, there are two main features for sound pressure distribution. When v is equal, sound pressure decreases as the distance increases. On the other hand, when the distance is equal, sound pressure decreases as the flow velocity increases.

### 3.3. Acoustic Receiving Features Influenced by the Flow Field inside the Sensor

In this subsection, by using the central ray (based on the ray acoustics) and the acoustic beam (or the receiver line, based on the wave acoustics), acoustic receiving features influenced by the flow field (from CFD) are also analyzed. 

#### 3.3.1. Receiving Features Obtained by the Central Ray

**Figure 14:**
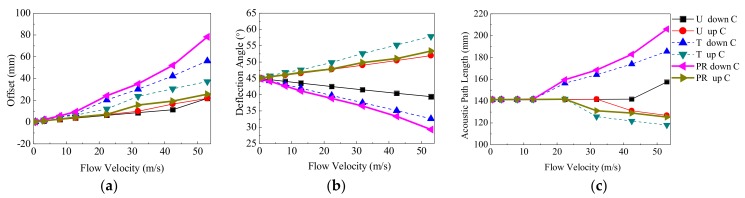
Receiving features of the central ray in different flow patterns. (**a**) Offset ($\Delta l_{dC}$, $\Delta l_{uC}$ ). (**b**) Deflection angle ($\alpha_{dC}$, $\alpha_{uC}$ ). (**c**) Length of ray path ($l_{dC}$, $l_{uC}$ ).

Figure 14 shows the receiving features of the central ray in different flow patterns. In Figure 14, four sets of parameters including (U, T, PR profile), (downstream, upstream), (v), and ($\Delta l_{uC}$, $\Delta l_{dC}$, $\alpha_{uC}$, $\alpha_{dC}$, $l_{dC}$, $l_{uC}$) are shown. The subscripts d and u represent downstream and upstream, respectively. From Figure 14, the conclusions are as follows:

- When v increases, for all velocity profiles, the parameters of $\Delta l_{uC}$, $\Delta l_{dC}$, $\alpha_{dC}$, $l_{uC}$, and $l_{dC}$ increase, whereas $\alpha_{uC}$ decreases.
- When v is equal, $\Delta l_{dC}$ > $\Delta l_{uC}$ for the PR and T profiles. $\alpha_{dC}$ < $\alpha_{uC}$, and $l_{dC}$ > $l_{uC}$ for all profiles.
- When v is equal, for parameters of $\Delta l_{C}$, $\alpha_{C}$, and $l_{C}$, the sequence of the three velocity profiles are as follows: PR > T > U for $\Delta l_{dC}$ and $l_{dC}$; T > PR > U for $\Delta l_{uC}$ and $\alpha_{uC}$; U > T > PR for $\alpha_{dC}$; U > PR > T for $l_{uC}$.

#### 3.3.2. Receiving Features Obtained by the Acoustic Beam

**Figure 15:**
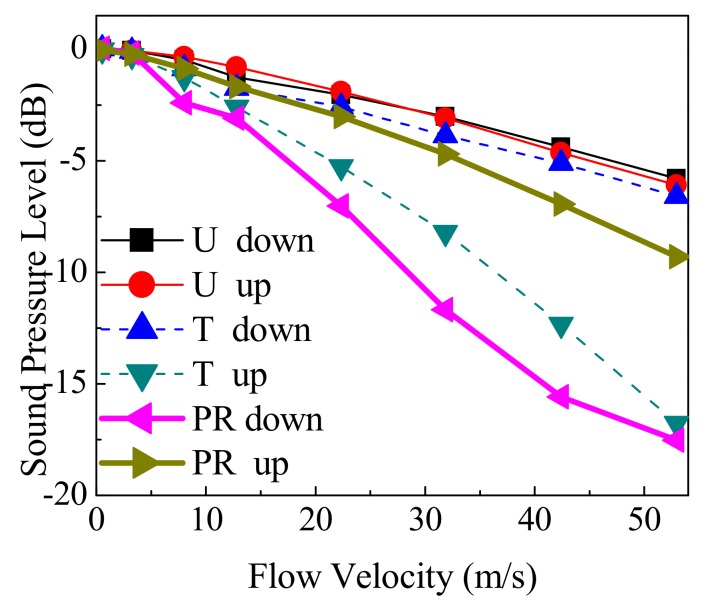
Sound pressure on the receiver for different flow patterns.

In this subsection, the receiving sound pressure is analyzed. Figure 15 shows the change of the average sound pressure level $Lp_{a}$ on the receiver using Equation (10).

(10) $$Lp_{a}=\frac{1}{L}\int_{L}Lp_{aR}dL$$

Here, $Lp_{aR}$ and L denote the sound pressure level on the receiver and the length of the receiver, respectively. In Figure 15, the average sound pressure level $Lp_{a0}$ of the stationary profile is used as a benchmark. Table 1 gives the relative deviation $\delta_{dev}$ between the $Lp_{a}$ and the $Lp_{a0}$ using Equation (11), which corresponds to Figure 15.

(11) $$\delta_{dev}=\frac{Lp_{a}-Lp_{a0}}{Lp_{a0}}\times 100\%$$

From Figure 15 and Table 1, the conclusions are given below:

- When v is small (such as 0.55 m/s < v < 12.82 m/s), the flow velocity and velocity profile have little effect on the sound pressure of the sound path. The $Lp_{a}$ of the three profiles is close, and the $\delta_{dev}$ is less than 3%.
- When v increases (for example, 12.82 m/s < v < 52.95 m/s), $Lp_{a}$ decreases. The $\delta_{dev}$ also increases, and the maximum deviation is 17%.
- When v is equal, for the downstream situation, the $Lp_{a}$ in descending order is U, T, and PR profiles. For the upstream situation, the sequence follows the order of U, PR, and T profiles. Both “the U and T profiles in the downstream section” and “the U and PR profiles in the upstream section” have the smallest deviations.

According to Figure 4, Figure 5 and Figure 6, and Figure 10, Figure 11 and Figure 12, the interpretations for the receiving sound pressure change as follows. Firstly, the changes can be explained by the central ray deflection that passes through the flow field. Secondly, the flow field zone sequence through which the acoustic beam passes causes the opposite changes between downstream and upstream. Moreover, the sound pressure value sequence for each profile downstream is opposite to the flow velocity sequence in the M zone.

### 3.4. Flowmeter Performance Influenced by the Flow Field Using the Fastest Ray

**Figure 16:**
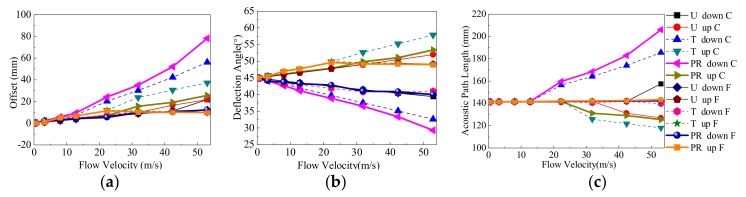
The receiving features of the central and fastest ray in different flow patterns. (**a**) Offset ($\Delta l_{dC}$, $\Delta l_{uC}$, $\Delta l_{dF}$, $\Delta l_{uF}$ ). (**b**) Deflection angle ($\alpha_{dC}$, $\alpha_{uC}$, $\alpha_{dF}$, $\alpha_{uF}$ ). (**c**) Length of ray path ($l_{dC}$, $l_{uC}$, $l_{dF}$, $l_{uF}$ ).

Figure 16 shows the receiving features of the central ray and the fastest ray in different flow patterns. In Figure 16, five sets of parameters including (U, T, PR profile), (downstream, upstream), (v), (the central ray, the fastest ray), and ($\Delta l_{C}$, $\alpha_{C}$, $l_{C}$, $\Delta l_{F}$, $\alpha_{F}$, $l_{F}$) are used for analysis. The conclusions are as below.

Firstly, the fastest ray is compared with the central ray. In a wide (or narrow) range of v, for example, 0 m/s < v < 42.41 m/s for the U profile (or v = 0.55 m/s for the T and PR profiles), the fastest ray and the central ray are the same one. So, the parameters of the fastest ray are same as those of the central ray. When v is not in the range described above (v is larger), the parameters for the central ray are greater than those of the fastest ray.

**Figure 17:**
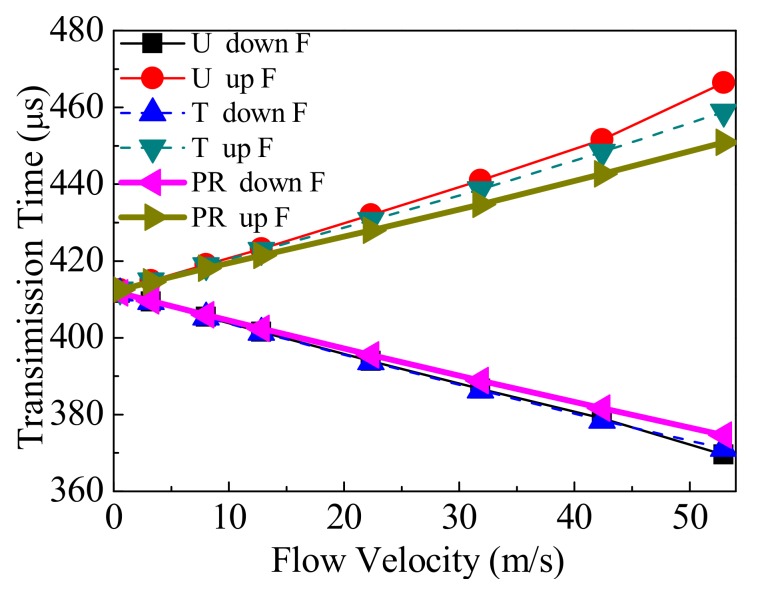
Transit time in different flow patterns.

Secondly, the transit time t is analyzed using the fastest ray. Figure 17 gives the transit time of the fastest ray in different flow patterns. From Figure 17, on the one hand, when v increases, $t_{uF}$ is also increased, but $t_{dF}$ is decreased. On the other hand, when v is equal, for each profile the descending order for $t_{dF}$ is PR > T > U, and that for $t_{uF}$ is U > T > PR.

Lastly, the instrument coefficient I that is deduced by Equations (12) and (13) is analyzed. It is found that $v_{F}$ shown in Equation (12) is not same as that of in Equation (1). Because the parameters in Equation (1) change based on the analyses in this paper, they need to be revised. So, Equation (12) is used. $v_{F}$ is compared with the inlet velocity v using Equation (13).

(12) $$\left\{ \begin{array}{l} v_{F}=\frac{\frac{l_{dF}}{t_{dF}}-\frac{l_{uF}}{t_{uF}}}{\cos\alpha_{dF}+\cos\alpha_{uF}}\\ t_{dF}=\frac{l_{dF}}{\left(c_{0}+v_{F}\cos\alpha_{dF}\right)}\\ t_{uF}=\frac{l_{uF}}{\left(c_{0}-v_{F}\cos\alpha_{uF}\right)} \end{array}\right.$$

(13) $$I=v_{F}/v$$

Table 2 shows the instrument coefficient for the three profiles. The conclusion is as follows:

- Firstly, in the range of 0.55~52.95 m/s for the T and PR profiles, the instrument coefficient decreases as the v increases. When v is equal, the descending order of I for each profile is U > T > PR. Compared with the U profile, the PR profile is closer to the T profile.
- Secondly, compared with the inlet velocity (used as a theoretical value), the maximum relative deviation of U, T, and SP profiles are 2%, −11%, and −17%, respectively.
- Thirdly, the deviation of the U profile between simulation and theoretical values is small, so the simulation scheme is feasible.
- Lastly, for the T and PR profiles (with a vortex near the transducer), the deviation is large between the simulation and theoretical values. That is a very important reason found in this work for many scholar seeking to improve the flowmeter performance by using various methods, including improving the velocity integral methods, waveform signal processing, and optimal arrangement of the multichannel, etc.

## 4. Validation by Experiment

**Figure 18:**
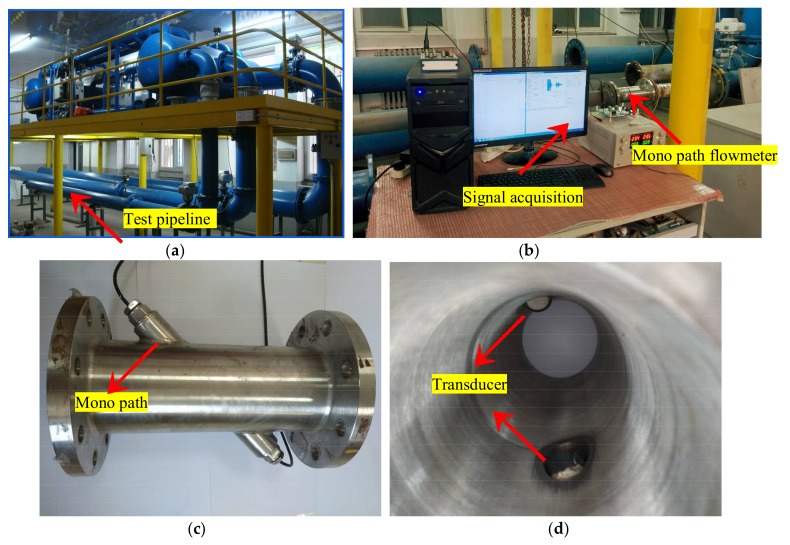
Experimental device and signal acquisition. (**a**) Gas flow device. (**b**) Signal acquisition. (**c**) Flowmeter with a single path. (**d**) Position of the transducer.

**Figure 19:**
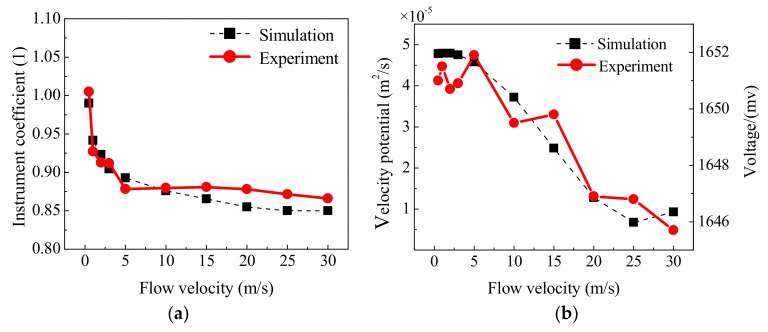
Comparison between the experiment and simulation. (**a**) Instrument coefficient. (**b**) Signal amplitude.

The simulation results can be compared with the real flow experiment. The experiment devices and program are as follows. Figure 18 shows the gas flow calibration facility in Tianjin Key Laboratory of Process Measurement and Control. A fan, standard gas turbine flowmeter, temperature and pressure sensors, computer control system, ultrasonic flowmeter, and straight pipe are contained in the device. For the device, the maximum velocity and precision are 42 m/s and 0.5%, respectively. The experimental environment employs normal pressure and temperature. The computer, data acquisition card, circuit board, and the ultrasonic flowmeter (limited to conditions, only mono path is selected) are included in the data acquisition system. According to the ultrasonic flowmeter verification regulations, a flowmeter with the required accuracy and repeatability is adopted. Ten velocities (Figure 19), three times for each measurement, and a 120-s sample time are selected for data acquisition. The signal emission interval between upstream and downstream is 5 ms, controlled by single chip microcomputer. The sampling frequency and sampling points are 4 MHZ and 3000 for each time, respectively [32,33,34]. The time delay and amplitude of receiving waveform are analyzed and compared with the simulation.

Figure 19a,b shows the instrument coefficient and the signal amplitude change with flow velocity for the PR profile, respectively. From Figure 19a, it is known that the relative deviation of the instrument coefficient between the theoretical value and simulation is 1–15%. The relative deviation between the theoretical value and experiment is 0–13%. The consistency between the simulation and the experiment is good. In addition, the instrument coefficient of the simulation and experiment for the PR profile exhibits a significant difference from the U and T velocity profiles (Table 2). It is illustrated that the velocity profile is the main factor that affects flowmeter performance. In Figure 19b, two vertical coordinates are used since there is no simulation on the transducer. The left coordinate is the velocity potential obtained by the simulation, the right coordinate is the voltage obtained by the experiment. By Figure 19b, for the signal amplitude, the velocity potential and voltage both decrease as the flow velocity increases, and the change trend is also consistent. It is noted that the geometry dimension and physical field are simplified during the simulation. Some differences can be caused between the simulation and the experiment.

## 5. Conclusions

A new hybrid scheme of flow-acoustic analysis fora gas ultrasonic flowmeter is proposed. In the scheme, the advantages of the three theories are utilized comprehensively. Firstly, CFD provide a background flow field close to the real flow. Secondly, the ray acoustics are better at capturing the time domain features of sound propagation. Thirdly, the wave acoustics can be well used to express the acoustic beam features generated by the piston surface source. It is noted that, some representative objects (including the central ray, ray beam, and fastest ray) or locations (including the center line of the acoustic path and the receiver line) are chosen for observing the flow pattern, sound propagation, and receiving. 

Based on the scheme, major conclusions regarding sound propagation and receiving are as follows. Firstly, the distributions of velocity profile and vortex intensity are asymmetric both on the sensor cross-section and acoustic path. Secondly, the sound pressure on the central line of the acoustic path and receiver is obviously influenced by the flow pattern. In particular, the central ray trajectory and the sound pressure on the central line of the acoustic path both change significantly when passing through the vortex zone near the transducer. Lastly, the parameters (such as offset, slope, path angle, and sound pressure, which are employed for quantification analysis) have significant differences among flow patterns.

At last, the instrument coefficient is determined. Compared with the inlet velocity, the maximum relative deviations of U, T, and PR profiles are 2%, −11%, and, −17%, respectively. The rationality of simulation is proved by the experiment.

Mainly from an academic perspective, the analysis scheme used in this work gives insight into the performance improvement of ultrasonic flowmeters for scholars. The scheme used in this work can help scholars find answers for various methods including velocity integral methods, waveform signal processing, and the optimal arrangement of the multichannel, etc. 

However, due to the complexity of the ultrasonic flowmeter, the research scope in this article is limited, as described below. The average inlet velocity of the three flow profiles and two-dimensional geometry are considered. The transducer diameter, transducer position, and ultrasonic frequency are fixed. Waveform signals, laminar flow and transition flow regimes are not considered. Therefore, other issues beyond the scope of this research still need to be studied. In the future, the more suitable schemes with respect to flow-acoustic coupling mechanism analysis, sensor design, and optimization of ultrasonic flow sensors should be explored.

## Figures and Tables

**Table 1:** Relative deviation of the sound pressure level on the receiver.

v (m/s)	Downstream (%)			Upstream (%)		
	PR	T	U	PR	T	U
0.55	0.03	0.02	0	−0.05	−0.04	−0.01
12.82	−3.1	−1.75	−1.26	−1.68	−2.61	−0.81
31.86	−11.68	−3.88	−3.02	−4.7	−8.21	−3.1
52.95	−15.03	−6.6	−5.2	−9.3	−16.77	−6.7

**Table 2:** Instrument coefficient.

v (m/s)	U	T	PR
0.55	1.00	1.00	0.95
3.24	1.00	1.00	0.90
8.04	1.00	0.99	0.88
12.82	1.00	0.98	0.87
22.35	1.00	0.97	0.85
31.86	1.00	0.94	0.85
42.41	1.01	0.93	0.84
52.95	1.02	0.89	0.83

## Abbreviations

CFD	Computational Fluid Dynamics
FEM	Finite Element Method
PR	Protrusion and Recess (of the transducer) flow velocity profile
SPL	Sound Pressure Level
T	Turbulent flow velocity profile
U	Uniform flow velocity profile
**Symbols**	
A	Transducer at the upstream section
a	Radiation surface radius of circular piston
B	Transducer at the downstream section
C	Central ray, subscript
$c_{0}$	Sound speed
*D*	Pipe diameter
d	Downstream, subscript
dσ	Fluid element
F	Fastest ray, subscript
f	Frequency
J	Vortex intensity
I	Instrument coefficient
k	Wave vector
k	Wave number
$k_{1}$	First component of wave number
$k_{2}$	Second component of wave number
L	Length of receiver
$L_{0}$	Ray direction vector
$Lp_{a}$	Average sound pressure level on receiver
$Lp_{a0}$	Average sound pressure level of stationary profile used as a benchmark
$Lp_{aR}$	Sound pressure level on receiver
$Lp_{1}$	Sound pressure level of analytical
$Lp_{2}$	Sound pressure level of simulation
l	Sound path length
$l_{\mathrm{AB}}$	Central line of acoustic path
$l_{AC}$	Straight line between A and C
$l_{AF}$	Straight line between A and F
$l_{C}$	Length of central ray
$l_{F}$	Length of fastest ray
n	Last discrete point number on ray trajectory
q	Ray position vector
$r_{0}$	Radius of point source
r	Sound propagation distance
$s_{1}$	Slope of line $l_{\mathrm{AB}}$
$s_{2}$	Slope of line $l_{AC}$
t	Transit time
u	Upstream, subscript
$V_{a}$	Vibration velocity amplitude
v	Flow velocity vector
v	Flow velocity
$v_{F}$	Flow velocity obtained by fastest ray
$z_{g}$	Far-near field demarcation of piston transducer
z	Axial coordinates on transducer axis
α	Sound path angle (the angle between the acoustic path and the pipeline axis)
β	Coefficient, β = 1
Δl	Offset at receiving position
$\Delta l_{path}$	Offset al.ong ray trajectory
$\Delta s_{path}$	Slope along ray trajectory
$\delta_{dev}$	Relative deviation between the $Lp_{a}$ and the $Lp_{a0}$
δ	Relative error between simulation and analytical value
ζ	Coefficient, ζ = −0.018
η	Coefficient, η = −1
θ	Angle between the direction of v and “the link line of the point source and the sound field point”
λ	Acoustic wavelength
$\rho_{0}$	Fluid density
ϕ	Acoustic velocity potential
ω	Normal component of rotating angular velocity for fluid element
ω	Acoustic angular frequency
